# Hypoxia-Inducible Factor-1alpha and MAPK Co-Regulate Activation of Hepatic Stellate Cells upon Hypoxia Stimulation

**DOI:** 10.1371/journal.pone.0074051

**Published:** 2013-09-10

**Authors:** Yueqin Wang, Yimin Huang, Fei Guan, Yan Xiao, Jing Deng, Huoying Chen, Xiaolin Chen, Jianrong Li, Hanju Huang, Chunwei Shi

**Affiliations:** 1 Department of Pathogen Biology, Tongji Medical College, Huazhong University of Science and Technology, Wuhan, Hubei, PR China; 2 Department of Parasitology, Tongji Medical College, Huazhong University of Science and Technology, Wuhan, Hubei, PR China; Juntendo University School of Medicine, Japan

## Abstract

**Background:**

Hepatic stellate cell (HSC) plays a key role in pathogenesis of liver fibrosis. During liver injury, hypoxia in local micro-environment is inevitable. Hif-1α is the key transcriptional regulation factor that induces cell’s adaptive responses to hypoxia. Recently, it was reported that MAPK is involved in regulation of Hif-1α activity.

**Aims:**

To explore whether Hif-1α regulates HSC activation upon hypoxia, and whether MAPK affects Hif-1α-regulated signaling cascades, thus providing new targets for preventing liver fibrosis.

**Methods:**

Hif-1α expression in livers of *Schistosoma* *japonicum* infected BALB/c mice was detected with western blot and immunohistochemistry. A rat cell line of HSC, HSC-T6, was cultured in 1% oxygen. HSC activation, including F-actin reorganization, increase of vimentin and α-SMA, was detected with western blot or immunocytochemistry. Cells were transfected with specific siRNA to Hif-1α, expression of activation markers, transcription of fibrosis-promoting cytokines, secretion of collagen I were detected with western blot, Real Time PCR and ELISA. Lysate from HSC-T6 cells pretreated with PD98059, a specific MEK1 pharmacological inhibitor, was subjected to detect Hif-1α ubiquitination and nuclear translocation with western blot and immunoprecipitation.

**Results and Conclusions:**

Hif-1α apparently increased in liver tissues of *Schistosoma* *japonicum* infected mice. 1% O_2_ induced F-actin reorganization, increase of Hif-1α, vimentin and α-SMA in HSC-T6 cells. Hif-1α Knockdown inhibited HSC-T6 activation, transcription of IL-6, TGF-β and CTGF and secretion of collagen I from HSC-T6 cells upon hypoxia. Inhibition of MAPK phosphorylation enhanced Hif-1α ubiquitination, and inhibited Hif-1α translocation into nucleus. Conclusively, Hif-1α and MAPK participate in HSC activation upon hypoxia.

## Introduction

Liver fibrosis is an important pathological feature of various chronic liver diseases, and is characterized by excessive deposition of extracellular matrix (ECM), especially collagen, in the liver [1,2]. Hepatic stellate cell (HSC) is the main cell source of ECM production when liver is injured by inflammation or mechanical stimulation. Quiescent hepatic stellate cells are vitamin A and lipid-storing cells, once activated, HSCs are transformed into myofibroblast-like cells (MFC), which leads to the loss of fat vacuoles and vitamin A, reorganization of cytoskeleton proteins, expression of α-smooth-muscle actin (α-SMA) and vimentin, then acquire the ability to synthesize plenty of collagen [3], secrete fibrosis-promoting cytokines such as TGF-β, CTGF and IL-6 and so on [4–7].

During liver damage and secondary inflammatory reaction, hypoxia in local micro-environment is inevitable. Hypoxia-inducible factor 1 (Hif-1) is the key transcriptional regulation factor which induces cell’s adaptive responses to hypoxic micro-environment and activates a number of hypoxia responsive genes. Hif-1 is composed of oxygen-regulated Hif-1α subunit and constitutively expressed Hif-1β subunit. Under normoxic conditions, Hif-1α undergoes continuous degradation through oxygen-dependent ubiquitination, keeping low concentration of Hif-1α. Upon hypoxia, the oxygen-dependent degradation pathway is inhibited and Hif-1α dimerizes with Hif-1β and enters the nucleus to bind with hypoxia-responsive elements (HRE) of target genes, thus, making cells survive in hypoxia [8,9]. Recently, it was reported that MAPK is involved in regulation of Hif-1α activity [10].

In this study, we aimed at exploring the function of Hif-1α in activation of hepatic stellate cells, which plays a key role in pathogenesis of liver fibrosis, and also the relationship of MAPK signaling pathway with Hif-1α-regulated signaling cascades in hepatic stellate cells. We firstly detected Hif-1α expression in liver tissues of *Schistosoma* *japonicum* infected mouse, which is regarded as a good model for infectious liver fibrosis and further used a rat cell line of HSC, HSC-T6, as a cell model, to investigate the effect of Hif-1α to HSC activation and also the effect of MAPK signaling to Hif-1α activity, thus providing new targets for preventing the progress of liver fibrosis. 

## Materials and Methods

### Animals

BALB/c female mice, 6–8 weeks old, were obtained from the Wuhan Institute of Biological Products, Wuhan, China. The experiment was approved by the Committee on Animal Research of Tongji Medical College, Huazhong University of Science and Technology. Mice were randomly divided into two groups: the infected group and the control group. Oncomelania snails infected with *S* *. japonicum* were purchased from Hunan Province Institute of Parasitosis Control and Prevention, Yueyang, China. *S* *. japonicum* cercariae were shed from the snails. Each anaesthetized mouse in the infected group was percutaneously infected with 25 cercariae through the shaved abdomen [11]. The mice were sacrificed at 6 weeks postinfection and samples of liver were collected.

### Cell culture, inhibitor treatment and hypoxia stimulation

HSC-T6 cell line, rat hepatic stellate cell line, was cultured at 37^o^C in room air (HF151, Heal Force, China) or in 1% oxygen in incubator (HF100, Heal Force, China) in Dulbecco’s modified Eagle medium, supplemented with 10% fetal bovine serum, 100U/ml penicillin and 100µg/ml streptomycin. Pretreatment of cells with 50µM PD98059 (S1805, Beyotime, China), a specific MEK1 pharmacological inhibitor, was performed as previously described [12].

### siRNA transfection

HSC-T6 cells were plated at a density of 2×10^5^ cells per well in 6-well plates. Eighteen hours post-seeding, cells were transfected with 100nM siRNA specific to Hif-1α (sc-45919, Santa-Cruz, USA) or nonspecific (NS) siRNA using Lipofectamine 2000 (Invitrogen, USA) following manual instructions.

### Western blot

Cells were collected at indicated time, washed twice with PBS and resuspended in lysis buffer (50mM Tris-HCl pH 8.0, 1mM EDTA, 250mM NaCl, 1% NP-40 and 0.5% Na-Deoxycholate). Cell lysates were shaken for 30 minutes on an orbital shaker at 4^o^C and centrifuged for 20 minutes at 12,000×g and the protein containing supernatant was collected. Protein concentration of cell lysates was estimated using a commercial kit (Bio-Rad, USA). SDS-PAGE and western blot were performed to determine the expression of Hif-1α (2015-1, Epitomics, USA), vimentin (bs-0756R, Bioss, China), α-SMA (A7607, Sigma, USA), phosphorylated MAPK (2219-1, Epitomics, USA), GAPDH (KC-5G5, Kangchen, China), H2AFX (10856-1-AP, Proteintech, USA) and β-actin (sc-47778, Santa-Cruz, USA).

### ELISA for collagen secretion

Detection of collagen I with ELISA was performed using COL-I enzyme linked immunosorbent assay kit (50R-E.1055R, Biovalue, China). Briefly, cell seeding, siRNA transfection were performed as described above. Supernatants were collected at indicated time and centrifuged at 1000×g for 20 minutes. Samples and enzyme conjugation solution were added into wells as the manufacturers’ instructions. Controls and samples were set in duplicate to assure consistency. OD value was read at 450 nm by ELISA reader (Biometra, Germany) and the concentration of collagen I was calculated according to the standard curve diagram. Data are presented as mean of collagen I concentration (μg/ml).

### RNA extraction and quantitative PCR

Total RNAs were extracted from cells using TRIzol Reagent (Invitrogen, USA) and 1µg RNA was reverse transcribed with ReverTra Ace ^®^qPCR RT Kit (TOYOBO, China) to obtain cDNA samples. Quantitative PCR was performed using Platinum®SYBR ®Green qPCR SuperMix UDG Kit (Invitrogen, USA) on the MyiQ Real-Time PCR Detection System (Bio-Rad, USA). All reactions were run in duplicates for three independent experiments. Cytokine-specific primers (Sangon Biotech, China) were listed as followed (Table 1). Gene expression was determined using the relative quantification. The amount of cytokine mRNA relative to β-actin mRNA was expressed as 2^-△△CT^ × 100% (ΔCT= CT_Target_ - CT_β-actin_, △△CT =△CT_Test_ - △CT_Control_). CT is the fractional cycle number that reaches a fixed threshold. ΔCT is the difference between gene expression in treated cells and reference control cells [13].

**Table 1:** Primers of rat TGF-β, IL-6, CTGF and β-actin.

**Name**	**Sequences of primers**	**Length (bp)**
TGF-β	5’- ATGGTGGACCGCAACAAC -3’	211
	3’- TAAGGACCGCAATGGAAC -5’	
IL-6	5’- GAGTTCCGTTTCTACCTGG -3’	204
	3’- TGTCTTCCTCACCGATTCC -5’	
CTGF	5’- CTGGTCCAGACCACAGAG -3’	199
	3’- GATTTTAACGGTTCGGAC -5’	
β-actin	5’- GTTGACATCCGTAAAGACC -3’	200
	3’- GAGTGACAGGTGGAAGGT -5’	

**qPCR of cytokine mRNA**. Total RNAs were extracted from cells using TRIzol Reagent (Invitrogen, CA) and 1µg RNA was reverse transcribed with ReverTra Ace®qPCR RT Kit (TOYOBO, China) to obtain cDNA samples. Quantitative PCR was performed using Platinum SYBR Green qPCR SuperMix UDG Kit (Invitrogen, USA) on the MyiQ Real-Time PCR Detection System (Bio-Rad, USA). All reactions were run in duplicates for three independent experiments. Expression of cytokines was normalized to β-actin.

### Immunocytochemistry

HSC-T6 cells were plated at a density of 2×10^5^ cells per well on coverslips, then cultured at 37°C in room air or in 1% oxygen. Forty-eight hours post-seeding, cells were fixed in 4% paraformaldehyde for 15 minutes, permeabilized with 0.1% Triton X-100 in PBS for 10 minutes, and counterstained with antibodies against vimentin (2707-1, Epitomics, USA) or FITC-phalloidin conjugates (P2141, sigma, USA). The coverslips were mounted onto slides in Anti-fade Mounting Medium (Beyotime, China) and fluorescent images were visualized and captured using Olympus BX51 upright fluorescent microscope (Olympus, Japan).

### Immunoprecipitation

Immunoprecipitation (IP) experiments were performed as described previously [12]. Briefly, cells were lysed in Lysis Buffer B (50mM Tris-HCl pH 8.0, 2mM EDTA, 150mM NaCl, 100mM NaF, 10% glycerol, and 1% Triton X-100) with protease and phosphatase inhibitors. Cell extracts (1mg) were mixed with 1µg of anti-Hif-1α antibody (sc-53546, Santa-Cruz, USA) and incubated at 4°C overnight with continuous agitation. Protein A-Sepharose beads (Amershan Pharmacia Biotechnology, USA) were added and incubated at 4°C for additional 2 hours. The beads were washed three times with Lysis Buffer B adjusted to 0.5% Triton X-100. Precipitated proteins were eluted by boiling the beads in 2×SDS-PAGE sample buffer for 5 minutes. The samples were analyzed by western blot with anti-ubiquitin antibody (6686-1, Epitomics, USA).

### Immunohistochemistry

The formalin-fixed and paraffin-embedded liver tissues were cut into 4-µm sections and then deparaffinized routinely. The slides were heated in 10mM citrate buffer (pH 6.0) for antigen retrieval. After washing with PBS for three times, the slides were incubated with 3% H_2_O_2_ at room temperature for 10 minutes and then incubated with Hif-1α primary antibody (AP7759c, Abgent, USA) at 4°C overnight. The slides were washed with PBS and incubated with polyperoxidase-anti-rabbit IgG (Envision™, DAKO, China) at room temperature for 30 minutes. After washing, the slides were colored with 3, 3-diaminobenzidine and counterstained with haematoxylin [11].

## Results

### Expression of Hif-1α apparently increased in liver tissues of Schistosoma japonicum infected mouse

**Figure 1:**
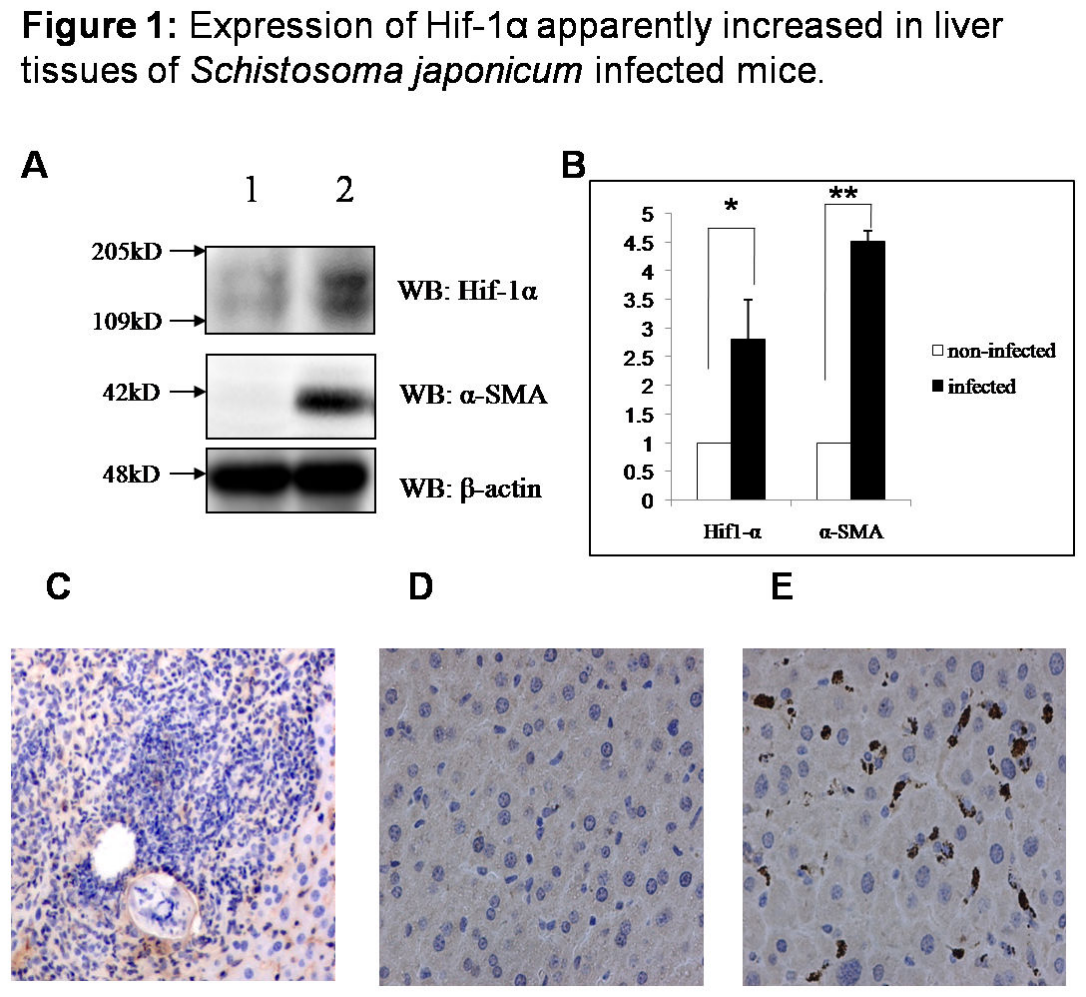
Expression of Hif-1α apparently increased in liver tissues of *Schistosoma* *japonicum* infected mice. BALB/c female mice, 6–8 weeks old, were percutaneously infected with 25 cercariae of *Schistosoma* *japonicum* through the shaved abdomen, sacrificed at 6 weeks post-infection and samples of liver were collected. The expression of Hif-1α and α-SMA in *Schistosoma* *japonicum* infected (n=3) and non-infected (n=3) mice liver was detected with western blot and immunohistochemistry. (A) 1: protein extract from the liver of non-infected Balb/C mice, 2: protein extract from livers of *Schistosoma* *japonicum* cercariae infected Balb/C mice. (B) Densitometric analysis (n=3). Data are mean ± SD. * : *P*<0.05, ** : *P*<0.01. (C) Acute inflammatory cell infiltration and granulomatous inflammation, surrounding eggs of *Schistosoma* *japonicum* . (D) Expression of Hif-1α in non-infected mice liver. (E) Expression of Hif-1α in infected liver.

In order to determine whether Hif-1α was induced in liver fibrotic tissues as liver was injured by infection, we firstly detected Hif-1α expression in liver tissues of *Schistosoma* *japonicum* infected mice, which is recognized as a good model for infectious liver fibrosis [11]. The expression level of Hif-1α in protein extract from the liver of *Schistosoma* *japonicum* infected mice was obviously higher than non-infected control mice (Figure 1A, 1B). In liver tissue section, apparent piecemeal changes of acute inflammatory cell infiltration and granulomatous inflammation, especially surrounding eggs of *Schistosoma* *japonicum* , were observed in the liver of infected mice, at 6 weeks postinfection (Figure 1C), as described elsewhere [11]. Expression of Hif-1α in liver section was significantly increased in infected mice (Figure 1E), when compared with non-infected mice (Figure 1D). With the increase of Hif-1α in the liver of *Schistosoma* *japonicum* infected mice, it was shown that expression of α-SMA, alpha-smooth muscle actin, an activation marker of hepatic stellate cells, was also increased in infected liver tissue (Figure 1A, 1B).

### 1% O_2_ induced apparent accumulation of Hif-1α and reorganization of F-actin in HSC-T6 cells

**Figure 2:**
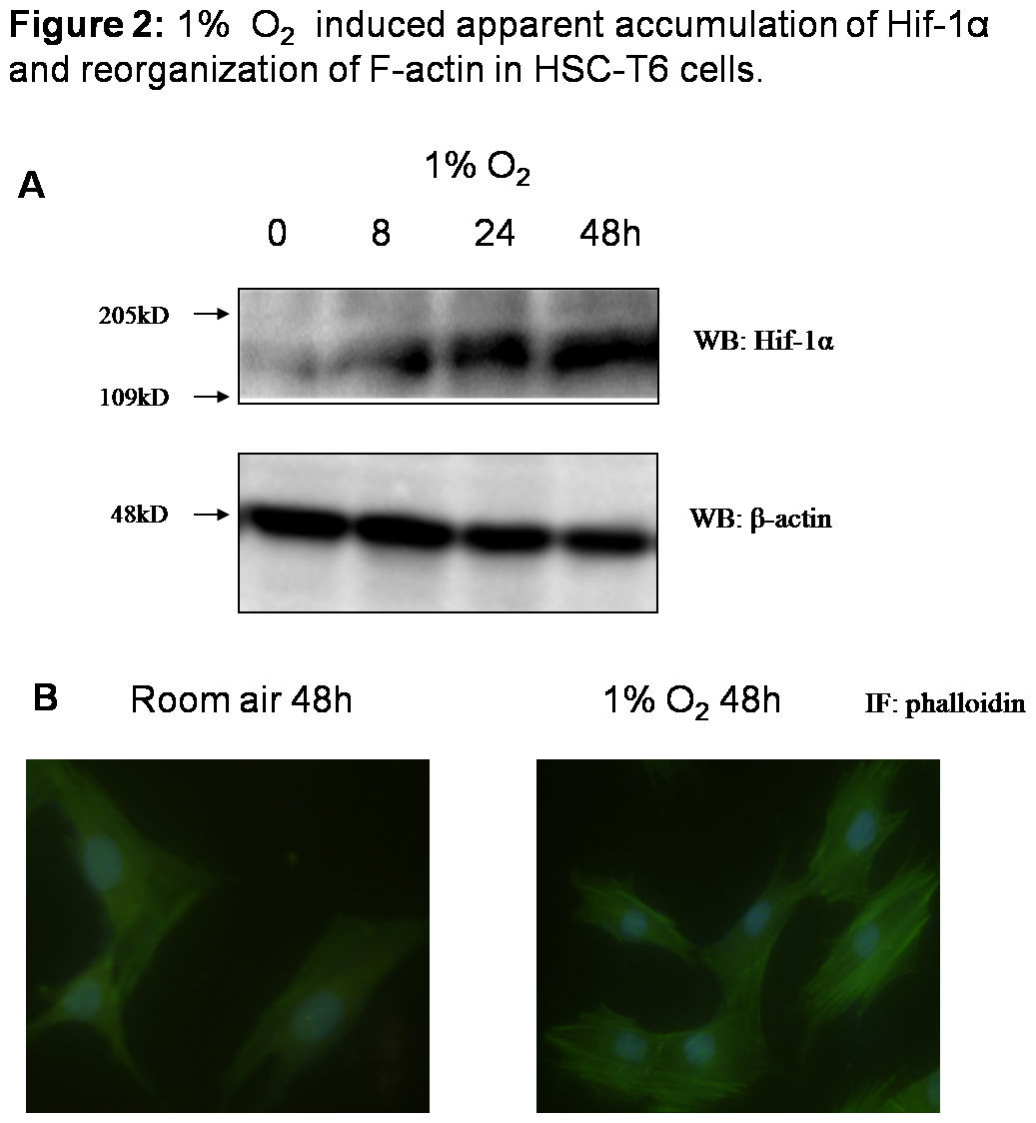
1% O_2_ induced apparent accumulation of Hif-1α and reorganization of F-actin in HSC-T6 cells. HSC-T6 cells, rat hepatic stellate cell line, were cultured in room air or in 1% oxygen. (A) Cells were collected at indicated time and cell lysates were subjected to detect Hif-1α and β-actin with western blot. (B) F-actin was stained using Fluorescein Isothiocyanate (FITC)-labeled phalloidin to capture F-actin of cells grown on the coverslips.

Hif-1α was apparently induced in HSC-T6 cells as cells were cultured in 1% oxygen at 24 hours and 48 hours (Figure 2A). Interestingly, it was found that hypoxic culture led to obvious elongation of cell shape and scattering of cells similar to the morphology of mesenchymal cells, indicating activated morphological changes of HSC-T6 cells upon hypoxia stimulation (data not shown). We therefore detected distribution of F-actin using immunocytochemistry. Compared with cells cultured under room air, cells exposed to hypoxia exhibited more apparent expression of F-actin, which was highly organized into fibers (Figure 2B).

### 1% O_2_ induced increased expression of vimentin and α-SMA in HSC-T6 cells

**Figure 3:**
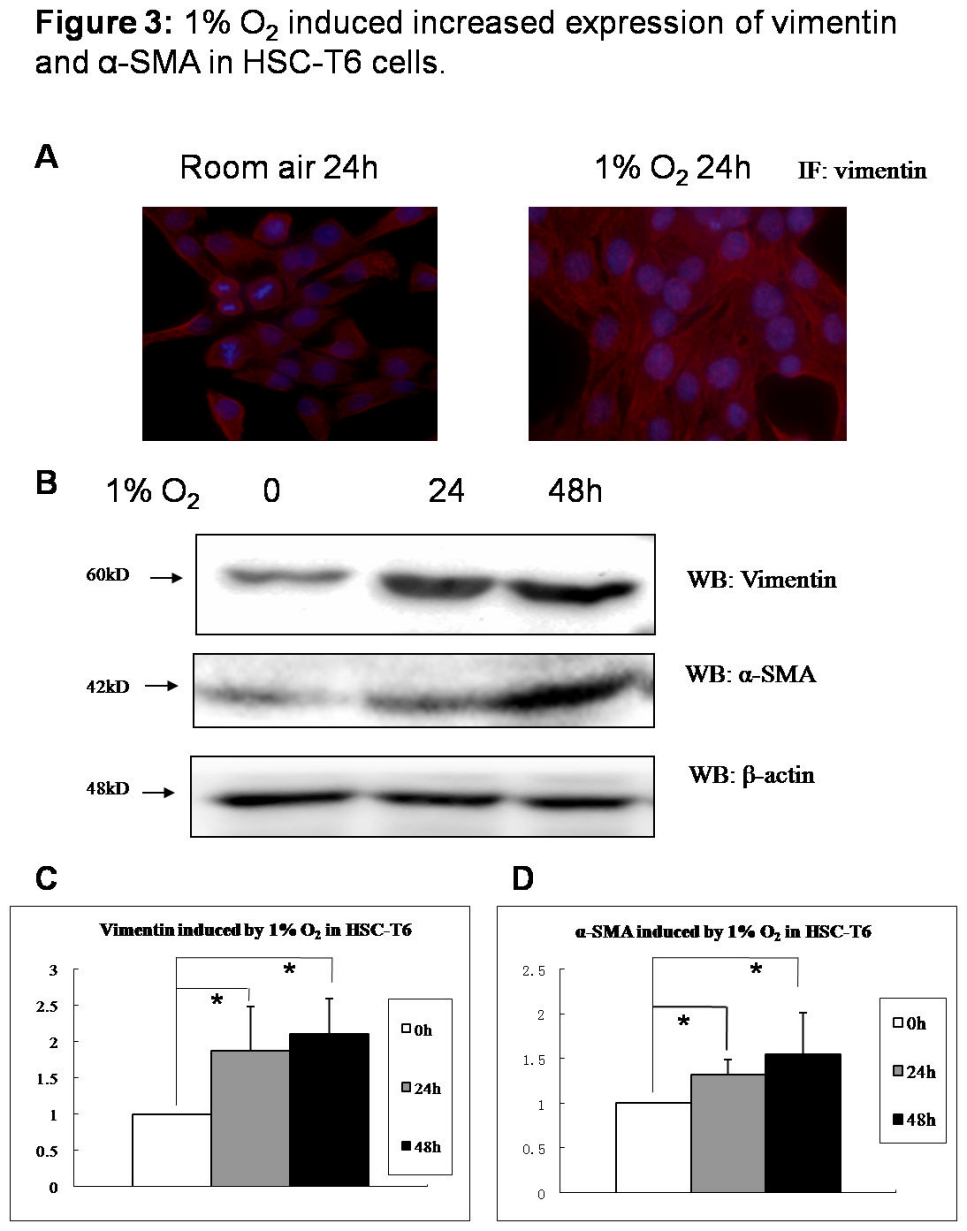
1% O_2_ induced increased expression of vimentin and α-SMA in HSC-T6 cells. HSC-T6 cells were cultured in room air or in 1% oxygen. (A) Vimentin expression in HSC-T6 cells grown on the coverslips at 48 hours post-seeding were co-stained with anti-vimentin (red) and DAPI (blue) by immunocytochemistry. (B) Cells were collected at indicated time and cell lysates were subjected to detect vimentin and α-SMA. Densitometric analysis was performed using pooled data from three such experiments. Data are mean ± SD. (C) vimentin; (D) α-SMA. * : *P*<0.05.

We further detected vimentin and α-SMA, which are recognized as activated markers in hepatic stellate cells. Increase of vimentin was observed in hypoxic HSC-T6 cells with immunocytochemistry and also western blot (Figure 3A, 3B and 3C). α-SMA expression was also more enhanced in HSC-T6 cells cultured in 1% oxygen, when compared to cells cultured in room air (Figure 3B and 3D). Apparent increase of vimentin and α-SMA indicated that HSCs were experiencing activation upon hypoxia stimulation, consistent with cell’s morphological changes and F-actin reorganization.

### Knockdown of Hif-1α inhibited induction of HSC-T6 activation markers upon cell’s hypoxia treatment

**Figure 4:**
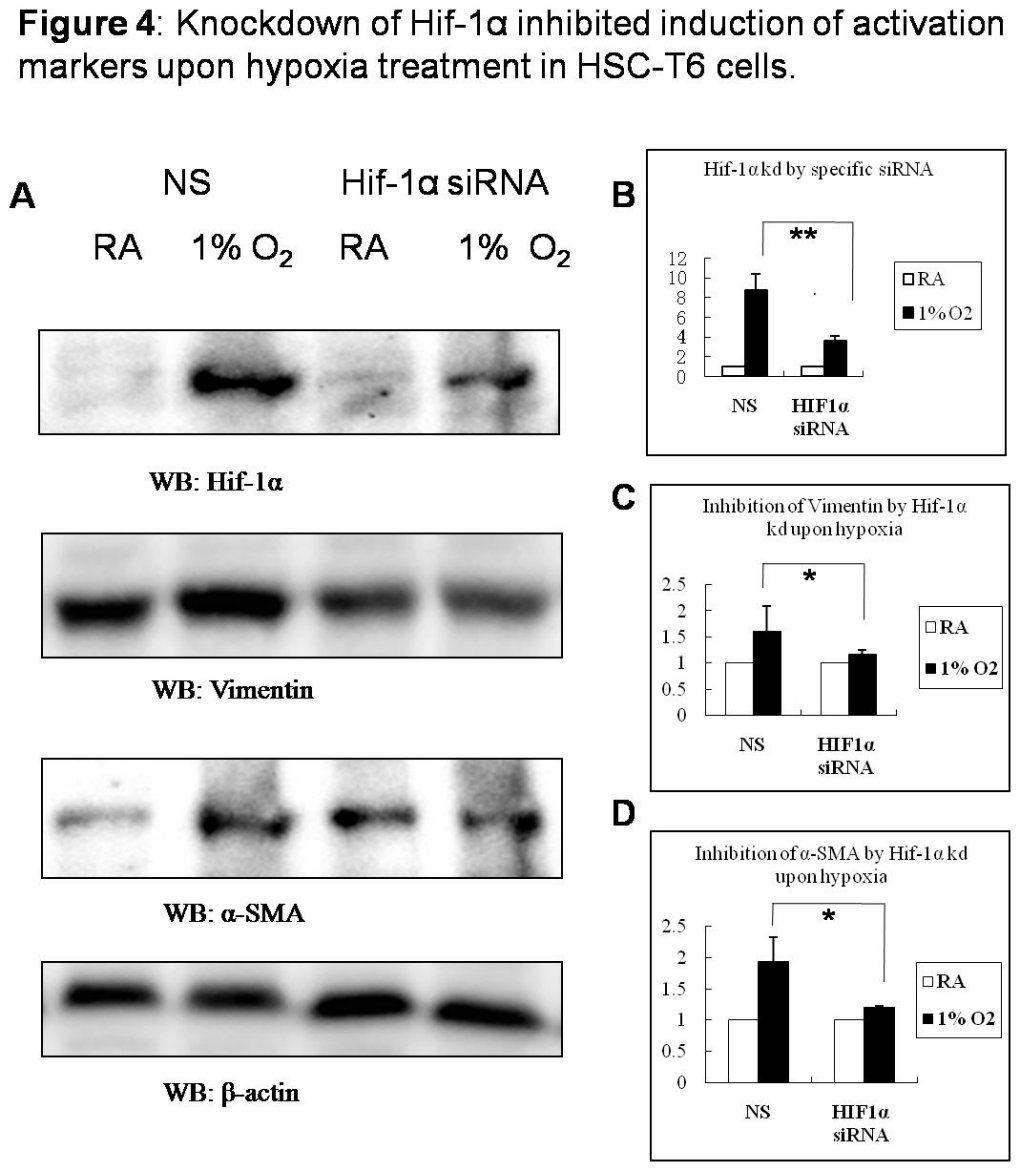
Knockdown of Hif-1α inhibited induction of activation markers upon hypoxia treatment in HSC-T6 cells. HSC-T6 cells in 6-well plates were transfected with either 100 nM Hif-1α siRNA or nonspecific (NS) siRNA for 24h and then cultured in room air or in 1% oxygen for 48h. (A) Cells were collected and cell lysates were subjected to detect Hif-1α, vimentin, α-SMA and β-actin with western blot; Densitometric analysis was performed using pooled data from three such experiments. Data are mean ± SD. (B) Hif-1α; (C) vimentin; (D) α-SMA. * : *P*<0.05, ** : *P*<0.01 (RA: room air, KD: knock down)..

To study whether Hif-1α plays an important role in activation of hepatic stellate cells upon hypoxia stimulation, Hif-1α siRNA was transfected into HSC-T6 to inhibit Hif-1α expression (Figure 4A, 4B). It was shown that upon hypoxia stimulation, expression of vimentin and α-SMA were inhibited as Hif-1α was silenced (Figures 4A, 4B, 4C and 4D). Accordingly, activated morphological changes of HSCs were not observed in Hif-1α siRNA-transfected cells as they were cultured in 1% oxygen (data not shown).

### Knockdown of Hif-1α inhibited transcriptional level of IL-6, TGF-β and CTGF and secretion of collagen I from HSC-T6 cells upon hypoxia treatment

**Figure 5:**
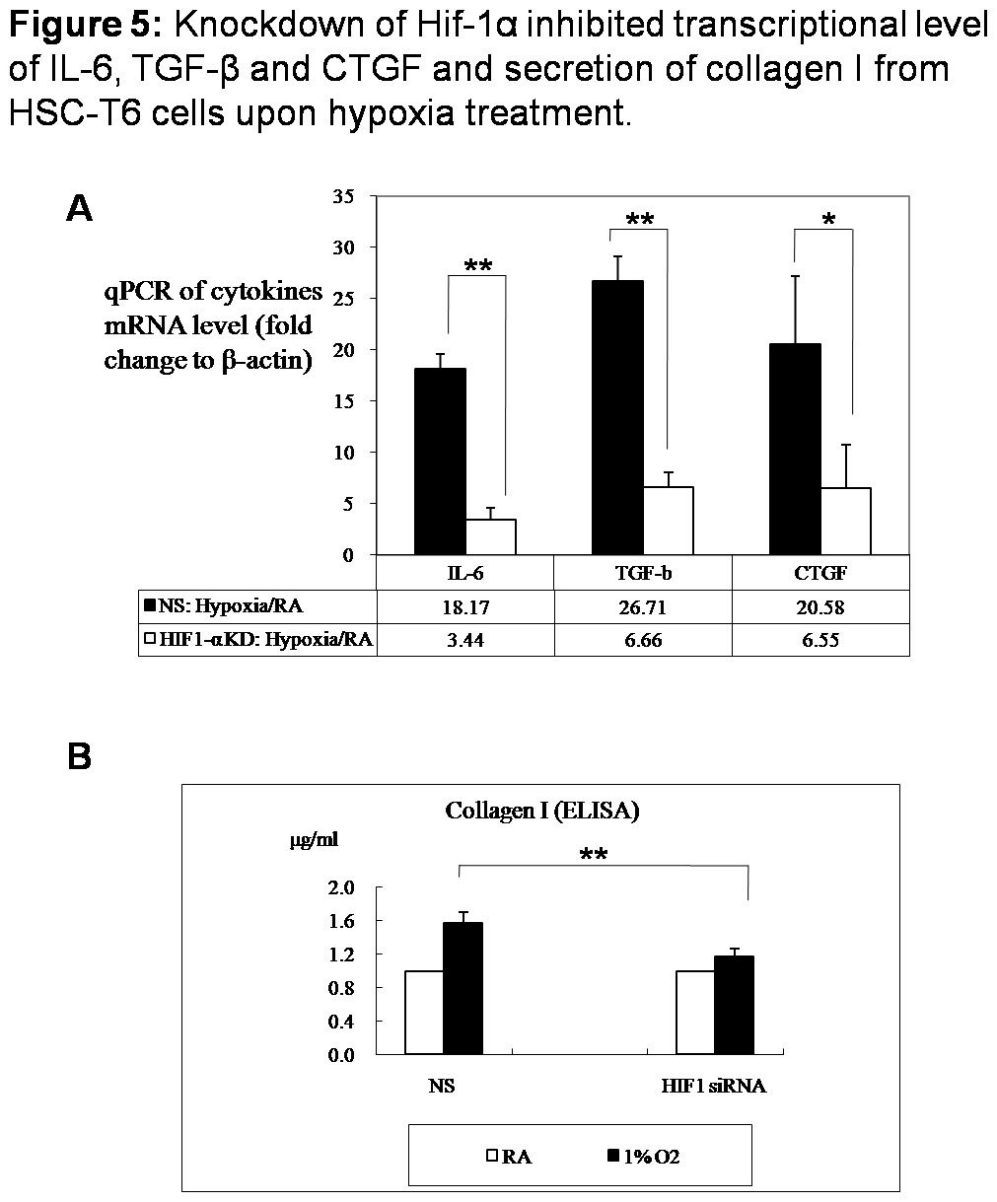
Knockdown of Hif-1α inhibited transcriptional level of IL-6, TGF-β and CTGF and secretion of collagen I from HSC-T6 cells upon hypoxia treatment. HSC-T6 cells in 6-well plates were transfected with either 100 nM Hif-1α siRNA or nonspecific (NS) siRNA for 24h and then cultured in room air or in 1% oxygen for 48h. (A) Cells were collected, total RNA was extracted and mRNA expressions of IL-6, TGF-β and CTGF were analyzed using qPCR. Expression of cytokines was normalized to β-actin. (B) Supernatant was collected and subjected to detect collagen I (μg/ml) with ELISA. Densitometric analysis was performed using pooled data from three such experiments. Data are mean ± SD. * : *P*<0.05, ** : *P*<0.01 (RA: room air, KD: knock down)..

Secretion of collagen I is the main function of activated hepatic stellate cells. Therefore we further detected transcriptional expression of fibrogenic cytokines TGF-β, IL-6 and CTGF and secretion of collagen I from HSC-T6 cells cultured in 1% O_2_ as Hif-1α expression was inhibited by siRNA. Transcriptional expressions of fibrogenic cytokines TGF-β, IL-6 and CTGF were detected with qPCR and expression of target genes was normalized with β-actin transcription. As shown in Figure 5A, TGF-β, IL-6 and CTGF were apparently induced as HSC-T6 cells were exposed to hypoxia, compared with cells, which were cultured in room air (RA). Hif-1α silencing by specific siRNA, significantly, but not totally, suppressed the increasing amplitude of TGF-β, IL-6 and CTGF transcription in HSC-T6 cells upon hypoxia treatment (Figure 5A). Secretion of collagen I was obviously increased in control group cells transfected with non-specific siRNA (NS group), as cells were cultured in hypoxia condition (collagen I concentration 1% O_2_/room air=1.573). Unlike NS group, collagen I secretion from HSC-T6 transfected with Hif-1α siRNA was not apparently increased, as cells were stimulated with 1% oxygen (collagen I concentration 1% O_2_/room air=1.176) (Figure 5B), which was consistent with the expression level of fibrogenic cytokines.

### 1% O_2_ induced MAPK phosphorylation in HSC-T6 cells and inhibition of MAPK phosphorylation enhanced Hif1-α ubiquitination, inhibited Hif-1α translocation into nucleus

**Figure 6:**
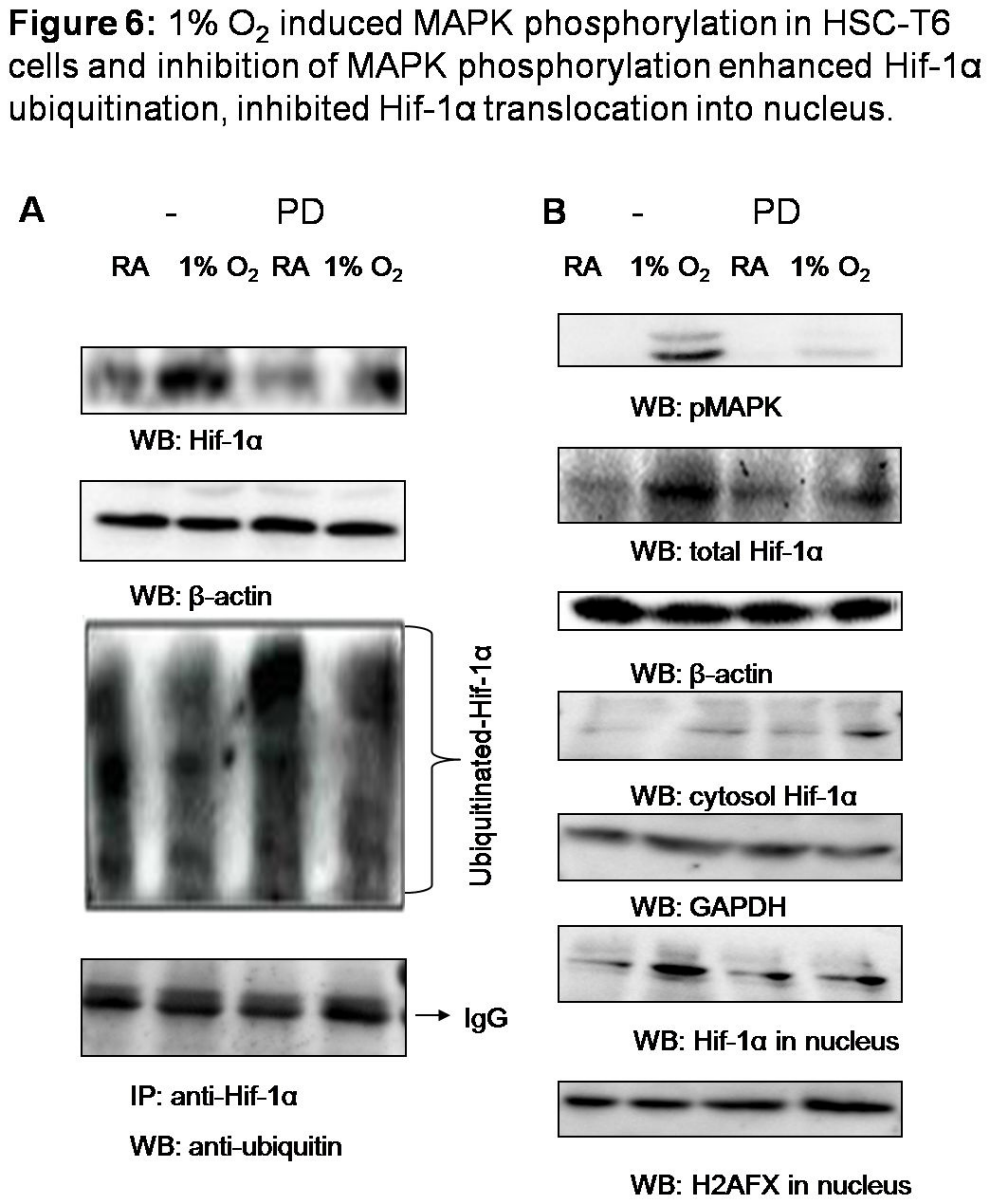
1% O_2_ induced MAPK pho*s*phorylation in HSC-T6 cells and inhibition of MAPK phosphorylation enhanced Hif-1α ubiquitination, inhibited Hif-1α translocation into nucleus. Cells were pretreated with PD98059 (50µM) or vehicle (DMSO) for 1h, and then cultured in room air or in 1% oxygen at 37°C for 15min. (A) 50µg of cell lysates was subjected to detect Hif-1α and β-actin with western blot and 1mg of lysates was immunoprecipitated with anti-Hif-1α, followed by immunoblotting with anti-ubiquitin. (B) Cells were collected and protein samples extracted from total lysates, cytoplasm and nucleus were subjected to detect phosphorylated MAPK, Hif-1α, β-actin, GAPDH and H2AFX with western blot.

As cells were exposed to hypoxia, it is interesting to find that MAPK phosphorylation was promptly induced in cells (Figure 6B). To test whether MAPK resides upstream or downstream of Hif-1α-regulated signaling cascades, we employed PD98059, a specific pharmacological inhibitor of MEK1 (the upstream kinase of MAPK), to block MAPK activation [13], and then assessed Hif-1α activity in HSC-T6 cells. It was shown that blocking of MAPK phosphorylation apparently inhibited Hif-1α accumulation in cells (Figure 6A, 6B). Cytosol synthesized Hif-1α was either continuously degraded through oxygen-dependent ubiquitination mechanism under normoxia, or translocated into nucleus to act as transcriptional factor upon hypoxia. Hif-1α ubiquitination and distribution of Hif-1α in total cell extract, cytoplasm and nucleus were further detected. As expected, blocking of MAPK phosphorylation with PD98059 enhanced Hif-1α ubiquitination and suppressed the increase of Hif-1α in cells upon hypoxic stimulation (Figure 6A). Although PD98059 pretreatment inhibited Hif-1α accumulation in cells, however, the level of cytosol Hif-1α was apparently increased in PD98059 pretreated cells, without increase of nuclear Hif-1α (Figure 6B), which indicated that blocking of MAPK phosphorylation resulted in the retention of Hif-1α in cytoplasm and the suppression of Hif-1α translocation into nucleus.

## Discussion

Live fibrosis is regarded as the excessive accumulation of extracellular matrix (ECM) proteins including collagen that occurs in most types of chronic liver injury, which leads to derangement of the architecture, portal hypertension and cirrhosis. Recent research indicated that hepatic fibrosis might be reversed when etiology is removed, synthesis of ECM is efficiently inhibited or degradation of ECM is promoted [1,2]. Therefore, promotion of hepatocyte regeneration and suppression of cells and cytokines involved in ECM production are major strategy in treatment of hepatic fibrosis. Understanding about pathogenesis of hepatic fibrosis is helpful to search for new targets to inhibit the progress of hepatic fibrosis.

Hypoxia in local micro-environment plays an important role in the pathogenesis of different diseases like tumors. Hif-1 is the key transcriptional regulation factor and activates a number of hypoxia responsive genes, thus making cells survive in hypoxia [5]. Hif-1 is a heterodimer consisting of an α subunit Hif-1α and β subunit Hif-1β, and Hif-1α is highly regulated by micro-environmental oxygenation, whereas Hif-1β is constitutively expressed. Most of previous studies focused on the role of Hif-1α in the pathogenesis of tumors and it was found that Hif-1α is over-expressed in most malignant solid tumors, and regulates many genes involved in angiogenesis, invasion and drug resistance [14–17]. Recently, it was reported that Hif-1α has also profound effect on liver fibrosis through regulating expression of genes for angiogenesis and collagen synthesis [18], but the regulatory mechanisms of Hif-1α in liver fibrosis is still unclear.

In present study, we firstly detected the expression of Hif-1α and α-SMA, alpha-smooth muscle actin, activation marker of hepatic stellate cells, in liver tissues of *Schistosoma* *japonicum* infected mice, which is regarded as a good model for infectious liver fibrosis. It was found that expression of Hif-1α and also α-SMA were significantly increased in the liver of *Schistosoma* *japonicum* infected mice, which point out that hypoxic micro-environment will be formed during liver damage and secondary inflammatory reaction caused by infection and hepatic stellate cells will be simultaneously activated. It is notably to notice that positive expression of Hif-1α mostly focus on non-hepatocytes, seemingly on interstitial cells. Whether those Hif-1α-positive cells are mainly activated hepatic stellate cells, needs still further investigation. The research performed in *Schistosoma* *japonicum* infection animal illustrated that Hif-1α is activated in liver infection and might be an important regulatory factor in liver fibrosis.

To explore the function of Hif-1α directly to the activation of hepatic stellate cells, which are main executors in liver fibrosis, a rat hepatic stellate cell line, HSC-T6 was used as cell model and stimulated with 1% O_2_. Upon hypoxia, reorganization of F-actin, increase of vimentin and α-SMA, indicated that HSCs were activated and phenotypic conversion happened. As Hif-1α was silenced with specific siRNA, it was shown that HSC-T6 activation was inhibited, without an increase of vimentin and α-SMA, which connects to focal adhesions and is essential to HSC activation, movement and migration [19,20].

Once HSCs are activated, HSCs produce pro-angiogenic cytokines such as VEGF, PDGF, Ang-1, VEGFR and also pro-fibrotic cytokines like TGF-β, important for angiogenesis and collagen synthesis [21–23]. We therefore detected transcription of fibrogenic cytokines TGF-β, IL-6 and CTGF and also collagen secretion. It was shown that Hif-1α silencing by specific siRNA suppressed the increasing amplitude of TGF-β, IL-6 and CTGF transcriptional expression in HSC-T6 cells and also collagen I secretion from cells upon hypoxia treatment. Recent researches indicated that, upon hypoxia stimulation, activated HSCs autocrine TGF-β1, trans-differentiate into myofibroblast-like cells through TGF-β1/Smad signaling pathway and promote production and deposition of ECM, resulting in hepatic fibrosis [24]. CTGF (connective tissue growth factor) is a lately reported cytokine, which is related with tissue fibrosis. CTGF functions downstream of transforming growth factor (TGF)-beta, driving cellular proliferation, increased extracellular matrix (ECM) accumulation and fibrosis [25]. IL-6 is a pro-fibrogenic and pro-inflammatory cytokine that activates and promotes HSCs proliferation via p38 MAPK [26] and α-SMA expression [6]. In current work, it was found that, although transcription of fibrogenic cytokines is dramatically suppressed by Hif-1α silencing in HSC-T6 cells upon hypoxia treatment, however, their expression still increased slightly. Hif-1α might be not the exclude factor regulating activation and function of HSC, however, researches in hepatic stellate cell line indicated that Hif-1α plays an important role in activation and function of hepatic stellate cell upon hypoxia stimulation.

Recently, it was reported that a variety of signaling pathways are involved in regulating Hif-1α at different levels. Phosphatidylinositol 3-kinase (PI3K)/Akt signaling pathway have been suggested to participate in both Hif-1α transcriptional activity and synthesis, while mitogen-activated protein kinase (MAPK/ERK) signaling pathway induces Hif-1α transcriptional activity and c-Jun protects Hif-1α from degradation leading to stabilization and cellular accumulation [27–29]. Despite of recent rapid advance in understanding the interactions between various pathways contributing to regulate Hif-1α, the links in regulating Hif-1α activity of liver fibrosis remain to be answered, since Hif-1α activity was proposed to be tissue specific [30]. In present work of hepatic stellate cells, induction of PI3K/Akt activation was not observed, however, rapid phosphorylation of ERK1/2 with the increase of Hif-1α under hypoxia stimulation were captured. It was found that MAPK phosphorylation stimulated by hypoxia is essential to Hif-1α activity, including attenuation of Hif-1α ubiquitination and promotion of Hif-1α nuclear translocation, so that Hif-1α enters the nucleus to act as transcriptional factor and regulating cell survival in hypoxia. Whether MAPK activation determines Hif-1α-regulated cascade, including Hif-1α stability, activity and also the synthesis and function of Hif-1α targeting genes, such as *vegf* [31], or MAPK is also a downstream target of Hif-1α with reverse regulatory effect, the detailed mechanism needs to be illustrated with further research.

Conclusively, it was shown in present study that Hif-1α was induced in liver tissues of *Schistosoma* *japonicum* infected mice, Hif-1α regulated the activation of hepatic stellate cells upon hypoxia stimulation and MAPK signaling mechanism was involved in regulation of Hif-1α regulated cascade, which provides us new targets to inhibit the development and progress of hepatic fibrosis.
